# Advantage of Using 8K Ultra-High-Definition Television System for Kasai Portoenterostomy for Biliary Atresia

**DOI:** 10.1055/s-0040-1721466

**Published:** 2021-01-27

**Authors:** Hisayuki Miyagi, Daisuke Ishii, Masatoshi Hirasawa, Tatsuya Shonaka, Yasuo Sumi, Nobuyoshi Azuma

**Affiliations:** 1 Division of Pediatric Surgery, Department of Surgery, Asahikawa Medical University, Asahikawa, Hokkaido, Japan; 2 Division of Gastrointestinal Surgery, Department of Surgery, Asahikawa Medical University, Asahikawa, Hokkaido, Japan; 3 Department of Vascular Surgery, Asahikawa Medical University, Asahikawa, Hokkaido, Japan

**Keywords:** biliary atresia, 8K ultra-high-definition television system, Kasai portoenterostomy, Kasai procedure

## Abstract

Kasai portoenterostomy (KPE) is currently the first-line treatment for biliary atresia. Many pediatric surgeons have reported that the dissection of the fibrous remnant at the porta hepatis is one of the most important components of this procedure. Furthermore, laparoscopic portoenterostomy is being increasingly used to treat biliary atresia.

An advantage of laparoscopic surgery is that surgeons can more easily identify microbiliary ducts, owing to the magnification. We report the case of a 61-day-old girl on whom we performed an exploratory laparotomy and diagnosed type III biliary atresia using intraoperative cholangiography. For the first time, we performed an open KPE using an 8K ultra-high-definition television system. This allowed us to clearly view the porta hepatis and to successfully perform the portoenterostomy.

## Introduction

The first successful surgical procedure for noncorrectable biliary atresia was reported in 1959 by Kasai et al. 1 More recently, Davenport et al reported that the 10-year native liver survival estimate was 40% in England and Wales, 2 Chardot et al reported the 10-year actuarial survival with a native liver to be 29% in France, 3 and Nio reported the 20-year native liver survival to be 49% in Japan. 4 Over the years, many technical modifications have been made to Kasai portoenterostomy (KPE), and many pediatric surgeons have reported that the dissection of the fibrous remnant at the *porta hepatis* is one of the most important components of this procedure. 5

Laparoscopy is now widely used in pediatric patients, and laparoscopic portoenterostomy (LPE) was first used to treat biliary atresia in 2002. 6 Some researchers have since reported favorable outcomes, 7 8 but others have reported that LPE is associated with a lower survival rate than open KPE when applied to native livers. 9

At our institution, we perform modified open KPE and often encounter patients in whom the bile outflow site cannot be easily observed during the resection of the fibrous remnant. Here, we report a case in which we performed open KPE and were able to confirm bile outflow from the dissection surface using an 8K ultra-high-definition (UHD) television system to view the *porta hepatis* , which allowed us to perform the portoenterostomy successfully.

## Case Report

**Fig. 1:**
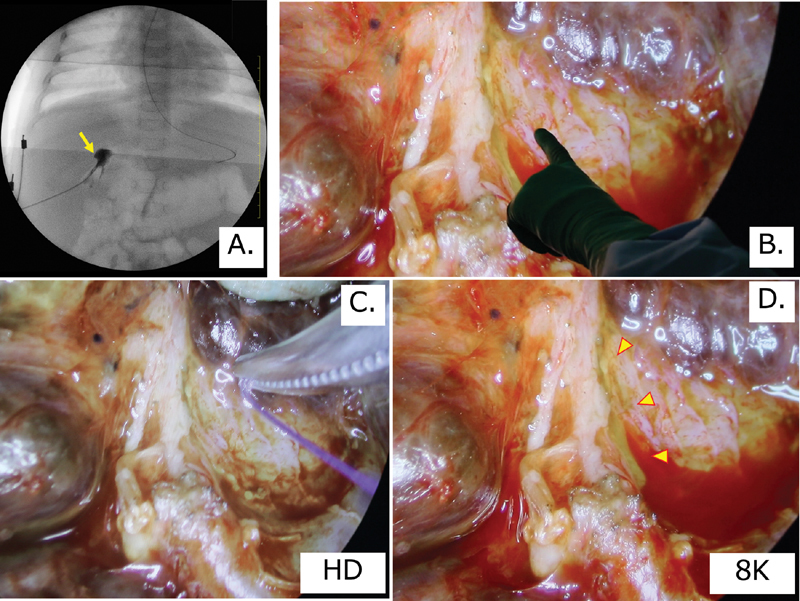
( **A** ) Intraoperative cholangiography was performed by inserting a tube from the bottom of the gallbladder (yellow arrow), but neither the common hepatic duct nor the common bile duct could be delineated, and compression resulted in leakage from the lateral aspect of the gallbladder. ( **B** ) The view of the surgical field was magnified on a 70-inch monitor and the site of bile outflow was confirmed by several pediatric surgeons (the fingers of the surgeon and outflow site are shown on the monitor). ( **C** ) View of the *porta hepatis* in the standard high-definition mode. Photograph comparing the resolution using 3–0 suture material (∼0.2 mm in diameter) in the standard high-definition mode. Bile outflow could not be visually confirmed. ( **D** ) View of the *porta hepatis* in 8K ultra-high-definition mode. Outflow of bile from a site near the right hepatic artery was confirmed (yellow arrows).

The patient was a 61-day-old girl. She was born at a gestational age of 37 weeks and had a birth weight of 2.41 kg. She had a slightly high galactose concentration on mass screening for congenital metabolic abnormalities at 4 days of age. She was examined at another hospital at 13 days of age, when she exhibited white feces and a high circulating direct bilirubin concentration. Her gallbladder could not be identified by abdominal ultrasonography; therefore, biliary atresia was suspected. At the age of 39 days, bile excretion was not observed on hepatobiliary scintigraphy, and hepatobiliary scintigraphy at the previous hospital had not confirmed excretion into the intestinal tract. Due to the novel coronavirus pandemic and the circumstances of the family, her referral to our hospital was slightly delayed. The patient was admitted to our department at the age of 57 days and underwent exploratory laparotomy at the age of 61 days, when she was diagnosed with type III biliary atresia (the type of extrahepatic biliary obstruction was determined using the Japanese Society of Pediatric Surgeons Classification 1 ) using intraoperative cholangiography ( Fig. 1A ) and underwent open KPE using the 8K UHD television system.

A right subcostal laparotomy was performed and intraoperative cholangiography was performed via the atrophied gallbladder. Only the gallbladder could be detected, and the contrast agent leaked from the lateral aspect under compression. A diagnosis of type III biliary atresia was made on the basis of the cholangiographic and gross findings, and open KPE was performed.

The connective tissue in the portal region was dissected using Metzenbaum scissors at the level of the hepatic capsule, from the medial side of the left portal vein toward the medial side of the right portal vein, while applying gauze saturated with warm physiological saline to aid hemostasis.

**Fig. 2:**
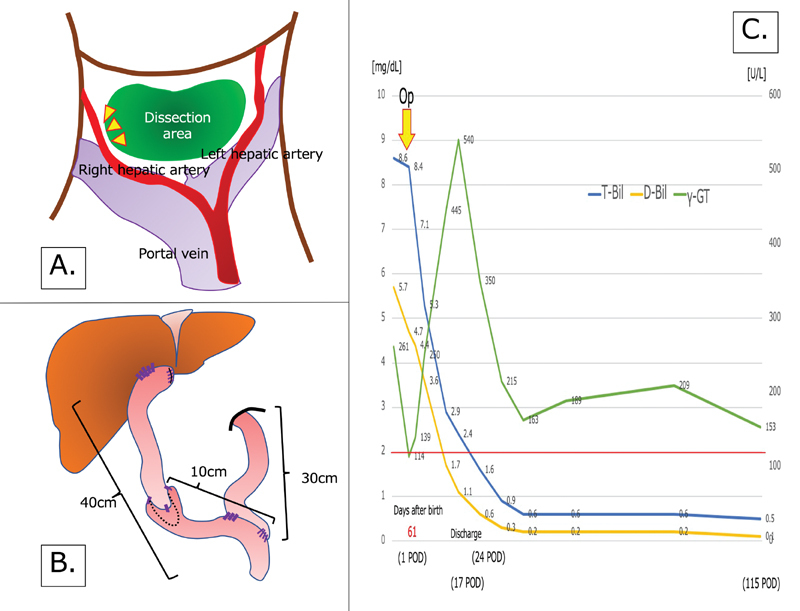
( **A** and **B** ) Schema of the surgery. ( **A** ) Outflow of bile was confirmed near the entrance of the right hepatic artery. ( **B** ) Schema of the biliary tract reconstruction using an artificial enteric valve. At our institution, we separate the jejunum 30 cm from the ligament of Treitz, such that the limb for a potential liver transplant is 40 cm long. Then, the limb is elevated to the *porta hepatis* , through the front of the transverse colon (ante-colic Roux limb route). ( **C** ) Trends in laboratory data. Graph showing changes in the circulating T-Bil and D-Bil concentrations, and gamma-glutamyltransferase activity over time. D-Bil, direct bilirubin; POD, postoperative day; T-Bil, total bilirubin.

At that time, the site of dissection was confirmed by examining the portal region using the camera of the 8K UHD laparoscopic system, and the biliary outflow site was examined after dissection. Bile outflow near the right hepatic artery was observed in 8K UHD mode ( Figs. 1B , D and 2A ), but was not visible in high-definition mode ( Fig. 1C ).

The jejunum was separated at a point 30 cm from the ligament of Treitz, an ascending limb of 40 cm in length was elevated in front of the colon, and portoenterostomy was performed. The jejunum was anastomosed to the *porta hepatis* using 5–0 PDS II, which sufficiently covered the biliary outflow site ( Fig. 2B ).

The biliary tract was reconstructed using the Roux-en-Y procedure, with the creation of a Nakajo-type enteric antireflux valve at a point 10 cm toward the liver from the Roux-en-Y limb and the Roux-en-Y limb was secured at an acute angle by suturing the seromuscular layer, to prevent food reflux. We performed an antireflux plasty using the intussuscepted valve Roux-en-Y procedure, according to the technique performed at Tohoku University Hospital. 2

The postoperative course was uneventful. Oral feeding was initiated on postoperative day (POD) 3, and the drain was removed on POD 6. Steroid administration was started, according to the protocol of the Japanese Biliary Atresia Society, on POD 7, and the patient was discharged on POD 17 with a total bilirubin concentration of 2.4 mg/dL. Thereafter, the patient was followed up on an outpatient basis, involving weekly attendance at the clinic during the immediate period following discharge, and once monthly visits when her blood parameters had normalized. She was initially followed up by both a pediatric surgeon and a pediatrician, but in the longer term she is being monitored principally by a pediatric surgeon. At the time of writing (POD 115), no cholangitis has been noted and her total bilirubin concentration is 0.5 mg/dL ( Fig. 2C ).

## Discussion

Many modifications have been made to the KPE technique over the years. In particular, the method used to determine the area and depth of resection of periportal connective tissue is an important issue. Nio et al 5 reported that the level of the hepatic capsule is appropriate as the resection line for the periportal connective tissue, and our department uses the same approach.

Previous studies have shown that the identification of microbiliary channels is often possible by magnifying laparoscopic images. 10 Laparoscopy may be inferior to microscopy in this respect, but it is not practical to insert and withdraw a microscope during LPE or open KPE. Therefore, we decided to use an 8K UHD laparoscopic system. An 8K UHD laparoscope was developed that was based on ultra-high-resolution 8K UHD imaging technology (7,680 × 4,320 pixels), which should enable physicians to perform safer and more efficient laparoscopic surgery. The 8K UHD laparoscopic camera weighed > 2 kg in 2014, but its weight was reduced to 370 g by Kairos Co. Ltd., and its commercial distribution as a medical device was approved in 2017. 11 Because the 8K laparoscope has 16 times higher resolution than a conventional high-vision laparoscope (1,920 × 1,080 pixels), it has become possible to identify fine blood vessels and membrane structures and distinguish important nerves that were previously difficult to identify visually. 11

We used the 8K UHD laparoscopic system to examine the hepatic portal region of a child with biliary atresia who was undergoing KPE, and observed slight bile outflow. Anastomosis was possible at the intended site, and her postoperative course was uneventful. Therefore, we expect the 8K UHD laparoscopic camera to be useful for laparoscopic and open surgery.

This procedure has two main limitations. First, the 8K UHD laparoscopic device contains a heated light source. Therefore, it should not be applied directly to the patient, its duration of use should be minimized, and the application of the light to a single site for a long period of time should be avoided. Second, although bile outflow was confirmed in the present case, it may be difficult to detect, depending on the type and stage of the disease. We do not recommend the use of the 8K system in cases in which bile outflow is clearly visible to the naked eye during surgery. However, by preparing an 8K UHD system preoperatively, we expect that bile outflow, which was previously invisible to the naked eye, may be rendered visible, and anastomosis can be facilitated. In the unlikely event that the 8K UHD system does not show bile outflow, the anastomotic site can be determined anatomically and empirically. In addition, long-term outcome data have yet to be collected, and therefore further cases should be studied over a longer period of time.

In conclusion, we performed open KPE using an 8K UHD laparoscope to examine the portal region. We confirmed outflow of bile from the resection surface and successfully performed open KPE using the laparoscopic system (8K Kasai procedure). This is the first report to describe the performance of this technique, which we expect to be useful for both LPE and open KPE.

## Notes

New Insights and the Importance for the Pediatric Surgeon We performed open Kasai portoenterostomy (KPE) using an 8K ultra-high-definition laparoscope to facilitate examination of the portal region and confirmed outflow of bile from the resection surface and successfully performed open KPE using this laparoscopic system (8K Kasai procedure). This is the first report to describe the performance of the 8K Kasai procedure, which we expect to be useful for both laparoscopic portoenterostomy and open KPE.
